# Hospital Patients and Medical Students

**Published:** 1887-03-19

**Authors:** John MacArthur


### Hospital Patients and Medical Students.

By John MacArthur.

Most of the larger hospitals of our larger cities are either directly or indirectly associated with medical schools, whether, as is generally the case in London, the hospital is an integral part of the school or, as in Scotland, there is a convention between the two organisations that the hospital is to be thrown open to students for purposes of clinical observation and research. It is of some importance, therefore, to inquire how far the fact bears upon the utility of the hospital, more especially as there exists in some quarters a feeling that the presence of medical students is prejudicial to its best interests, retarding the cure of the patients, and distracting the attention of the staff.

The main object of a hospital undoubtedly is to cure disease; but to this, in order to the adequate training of medical practitioners, it has been found necessary to superadd the provision of opportunities for the study and observation of actual disease. Does this second object in any measure deter from the satisfactory prosecution of the first.

The part which a hospital has to play in medical education is fundamentally that of a clinical school. There students *see* that of which they merely *hear* in the class-room. Hence, if they are to profit at all by their attendance, they must be brought into frequent contact with the patients. How then can the relations between the two be most satisfactorily maintained?

The standpoint from which the student views the question is, of course, that the more he can see of disease the better. He naturally takes advantage of every opportunity that offers of doing so, and he looks to his teachers to further his efforts as much as they can. That this zeal on his part in many cases retards, even prevents, the cure of the patient, has been seriously alleged. No doctor, it is said, would permit in his private practice that which he, to some extent, encourages in the hospital—the crowding round the bed; the hurried examination of diseased parts; the practice of all the different means of medical and surgical investigation. Such is the picture which the imaginative outsider generally draws of what goes on in our infirmaries, and the inference, to which he at once rushes, is that such circumstances cannot but be injurious, cannot but break in on the repose always so essential, cannot but harass the feelings of the sensitive, cannot but create in the minds of the nervous sick a dread of entering the hospital at all.

The outsider, however, has far too fertile an imagination. The main lines of the picture are perhaps correct, but there is far too much colouring. That the resulting evils are at all commensurate with what it implies, or indeed that they exist at all, except in a few extreme cases, will be at once and emphatically denied by all who have any real insight into hospital work. In every well-regulated hospital there are preserving influences of a powerful kind at work, which effectually restrain the enthusiasm of the student, and prevent him from doing any real injury.

The most important safeguard—and it is one which is an almost absolutely reliable one—lies in the common sense and prudence of the physicians and surgeons, under whose superveillance the student conducts his investigations. What medical man who has attained to the dignity of being a member of a hospital staff would permit those under his charge roughly to handle a dying man, or would encourage them closely to question and examine a delicate, nervous woman? Although there can be no general rule laid down as to when and how far a case should be used for educational purposes, it will invariably be found that in every hospital with the slightest pretence to being well regulated, no patient will be asked to submit to any examination or observation of this kind, unless his disease is capable of giving real information, or unless he is in such a physical condition that such observations would do no harm. In addition to this, it must also be remembered, despite the popular impression to the contrary, that as a class medical students are gentlemen, and that their own human instincts in such cases generally guide them aright. There is within them a feeling, born of culture and experience, which tells what to do, and what not to do.

Such, then, are some of the disadvantages which it is alleged the presence of medical students occasions in a hospital. We have endeavoured to show that they do not exist; but we have yet to deal with the other side, to point out some of the benefits which the presence of students invariably confers on such an institution. From them the hospital receives material services, and that apart from all question of the fees they pay,—and, after all, fees are not to be despised. In many cases the student undertakes a considerable share of the dressing, and in so doing he materially saves the time and trouble of the nurses, and enables them to do their other work more efficiently. When a case is allotted to him he is often able, if he be possessed of fair intelligence, to give information as to symptoms and progress, which may have an important bearing on treatment. In most cases, too, his attentions and society, so far from being irksome to the patients, are positively grateful. Their happiest hour is that in which the dreary monotony of inaction and pain is broken by the sight of bright faces, and by cheering words which bring them into contact with the outer world again.

Not only so, but the very presence of the student is an incentive to all connected with the hospital to do their work better. He gives a standing to it which both the staff and he do their very best to maintain. This is no mere theory; it is an actual fact. The most efficient hospitals in this country are those the wards of which are daily thronged by eager students.

It is often said that those who avail themselves of a charity have no right to complain of any little sacrifice which they may be called on to make in connection with their position. This is true; but the right is one which no right thinking practitioner or student in this country would care much to insist on.

After all, what the student does for the hospital is as nothing to what it does for him. It gives him an opportunity which, now that the old system of apprenticeship has died out, he could not otherwise have of observing actual disease, and of becoming familiar with its myriad forms. Without this he would be utterly incapable of undertaking that work to which he has devoted his life.

But there is still another service which the hospital does for the student, hardly less important. It is in its wards that he first learns what suffering is, that he first feels how noble a thing it is to strive to relieve it; and it is there that he makes the invaluable discovery that there are methods of treatment beyond the resources of pharmacopœia or surgery; that a winning smile, a kind word, or a gentle touch, if they cannot of themselves lessen pain, can do much to help the sufferer to bear it.

